# *Plasmodium falciparum* in Ancient Egypt

**DOI:** 10.3201/eid1408.080235

**Published:** 2008-08

**Authors:** Andreas G. Nerlich, Bettina Schraut, Sabine Dittrich, Thomas Jelinek, Albert R. Zink

**Affiliations:** * Academic Teaching Hospital München-Bogenhausen, Munich, Germany; † University of Manchester, Manchester, UK; ‡ Institute of Tropical Medicine, Berlin, Germany; 1 Current affiliation: Eurac, Bolzano, Italy.

**Keywords:** malaria, Pfcrt gene, ancient Egypt, letter

**To the Editor:** Malaria is a disease caused by parasites of the genus *Plasmodium.* The infection is transmitted to humans through the bites of female mosquitoes of the genus *Anopheles.* Four species of *Plasmodium* are pathogenic to humans, and each leads to different clinical features: *P. falciparum* causes severe malaria with undulating high fever (malaria tropica); *P. malariae, P. vivax,* and *P. ovale* cause less severe clinical courses of disease with the manifestations of malaria quartana (*P. malariae*) and malaria tertiana (*P. vivax* and *P. ovale*). Literary evidence for malaria infection dates back to the early Greek period when Hippocrates described the typical undulating fever (*1*), highly suggestive of plasmodial infection. Although it is believed that malaria widely affected early pre-Hippocrates populations, until now only 1 study, which used molecular analysis, clearly identified *P. falciparum* in a Roman infant dating back to the 5th century AD (*2*). Two other studies used molecular analysis to identify more recent plasmodial DNA in ancient human remains, i.e., from 100–400 years ago (*3*,*4*). A substantial number of nonspecific amplifications in these previous studies raised concerns as to the specificity of current molecular markers for ancient malaria (*3*,*4*).

In this report, we describe the unambiguous identification of ancient DNA (aDNA) for *P. falciparum* in ancient Egyptian mummy tissues from ≈4,000 years ago. We analyzed 91 bone tissue samples from ancient Egyptian mummies and skeletons. The Egyptian material derived from the Predynastic to Early Dynastic site of Abydos (n = 7; 3500–2800 BC), a Middle Kingdom tomb in Thebes West (n = 42; 2050–1650 BC), and various tomb complexes in Thebes West, which were built and used between the Middle and New Kingdom until the Late Period (n = 42; c. 2050–500 BC). All samples were first tested for *Plasmodium* spp. DNA by using the heminested PCR for the 18S rDNA primer targets usually used for malaria identification (*5*). Direct sequencing was performed on those with positive amplification products. Thereby, a high number of amplification products of various sizes (including the expected size) were detected. However, on sequencing, all amplicons provided nonspecific products. Consequently, in a second set, all material was tested for the *P. falciparum* chloroquine-resistance transporter gene (*pfcrt* gene) (*6*,*7*), which was also further characterized by direct sequencing.

In this second set of experiments, 2 of the 91 ancient Egyptian samples tested positive for the 134-bp fragment of the pfcrt region of *P. falciparum* (Figure). The specificity of the amplification was verified by sequencing, which showed 99% sequence concordance. The result was verified by parallel analysis in 2 independent laboratories; observations were fully concordant. The 2 positive samples originated from 2 different tomb complexes dating from the New Kingdom until Late Period (1500–500 BC). Each sample was obtained from adults who had osteopathologic evidence of chronic anemia. No positive results were found for the earlier samples from the Predynastic to Early Dynastic or Middle Kingdom periods.

**Figure:**
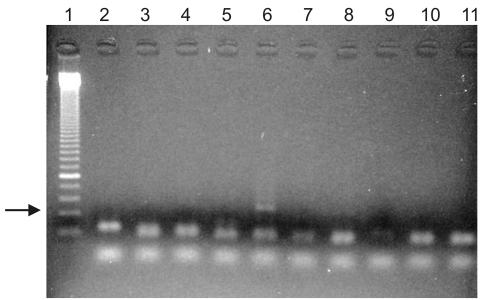
PCR amplification of a 134-bp fragment of ancient DNA of *Plasmodium falciparum* in Egyptian mummies. Lane 1, molecular marker; lanes 10 and 11, 2 negative controls. One (lane 6) of 8 samples shows a positive amplification product (arrow). Specificity of the product was verified by sequencing.

Previously, immunologic tests have been used to investigate the presence and incidence of malaria in ancient Egyptian mummies (*8*,*9*). Because >40% of all samples and 92% of samples from persons with bone lesions suggestive of chronic anemia tested positive for the *P. falciparum* histidine-rich protein-2 antigen, doubts as to the specificity of those tests have been raised.

Our study unambiguously identified *P. falciparum* aDNA in Egyptian mummy samples, thereby proving a specific infection by falciparum malaria in ancient Egypt. With respect to the infection incidence, our molecular analysis suggests a more realistic frequency than had been previously suggested by paleoimmunologic methods. Consequently, the aDNA analysis is superior with respect to the reaction specificity, so that the latter should not further be used for that purpose.

This report adds another infectious disease to the spectrum of paleomicrobiology in ancient Egypt, thereby further explaining the previously postulated influence of infectious diseases on the low life expectancy for ancient Egyptian populations (*10*). Molecular detection of pathogen aDNA can be used not only to identify a certain disease, but it may also provide information on disease frequency, evolutionary origin, and pathways.
